# Identification of Rab41/6d Effectors Provides an Explanation for the Differential Effects of Rab41/6d and Rab6a/a' on Golgi Organization

**DOI:** 10.3389/fcell.2016.00013

**Published:** 2016-03-01

**Authors:** Shijie Liu, Waqar Majeed, Tetyana Kudlyk, Vladimir Lupashin, Brian Storrie

**Affiliations:** Department of Physiology and Biophysics, University of Arkansas for Medical SciencesLittle Rock, AR, USA

**Keywords:** Golgi organization, Rab41/6d, effector, dynactin 6, syntaxin 8, Rab6a/a'

## Abstract

Unexpectedly, members of the Rab VI subfamily exhibit considerable variation in their effects on Golgi organization and trafficking. By fluorescence microscopy, neither depletion nor overexpression of the GDP-locked form of Rab6a/a', the first *trans* Golgi-associated Rab protein discovered, affects Golgi ribbon organization while, on the other hand, both Rab41/6d depletion and overexpression of GDP-locked form cause Golgi fragmentation into a cluster of punctate elements, suggesting that Rab41/6d has an active role in maintenance of Golgi ribbon organization. To establish a molecular basis for these differences, we screened for Rab41/6d interacting proteins by yeast two-hybrid assay. 155 non-repetitive hits were isolated and sequenced, and after searching in NCBI database, 102 different proteins and protein fragments were identified. None of these hits overlapped with any published Rab6a/a' effector. Eight putative Rab41 interactors involved in membrane trafficking were found. Significantly, these exhibited a preferential interaction with GTP- vs. GDP-locked Rab41/6d. Of the 8 hits, the dynactin 6, syntaxin 8, and Kif18A plasmids were the only ones expressing the full-length protein. Hence, these 3 proteins were selected for further study. We found that depletion of dynactin 6 or syntaxin 8, but not Kif18A, resulted in a fragmented Golgi apparatus that displayed a Rab41/6d knockdown phenotype, i.e., the Golgi apparatus was disrupted into a cluster of punctate Golgi elements. Co-immunoprecipation experiments verified that the interaction of dynactin 6 and syntaxin 8 with GTP-locked Rab41/6d was stronger than that with wild type Rab41/6d and least with the GDP-locked form. In contrast, co-immunoprecipitation interaction with Rab6a was greatest with the GDP-locked Rab6a, suggestive of a non-physiological interaction. In conclusion, we suggest that dynactin 6, a subunit of dynactin complex, the minus-end-directed, dynein motor, provides a sufficient molecular basis to explain the active role of Rab41/6d in maintaining Golgi ribbon organization while syntaxin 8 contributes more indirectly to Golgi positioning.

## Introduction

In mammalian cells, the Golgi apparatus plays a central role in membrane trafficking pathways. Often, it is organized into a juxtanuclear, ribbon-like structure composed of multiple Golgi membrane stacks. Each stack consists of a series of flattened, membrane-bound discs termed cisternae which on the basis of their resident enzymes and functions can be classified into four distinct regions: *cis*, medial, *trans*, and *trans*-Golgi network (TGN) (for reviews, see Wei and Seemann, 2010; Lowe, 2011). This highly organized structure is essential for modification and sorting of cargo proteins. Golgi ribbon organization is highly regulated and controlled, in part, by Rab proteins, the largest family of small Ras-like GTPases. Rab proteins act as molecular switches that recruit effectors in the GTP-bound state (for review, see Liu and Storrie, 2012).

Among the ~70 mammalian Rab proteins, Rab6a, Rab6a', Rab6b, Rab6c, and Rab41 constitute the Rab VI subfamily on the basis of sequence homology (Pereira-Leal and Seabra, 2001) and protein folding/surface charge exposure (Stein et al., 2012). Rab6a and a' are two closely related isoforms that differ in only 3 amino acids (Echard et al., 2000). They are ubiquitously expressed, present in equal amounts, and localize to the *trans* Golgi and TGN (Goud et al., 1990; Antony et al., 1992; Sun et al., 2007). They exhibit sufficiently similar biochemical and genetic properties that they are often collectively referred to as Rab6 (Echard et al., 2000). Unlike Rab6a/a', Rab6b is preferentially expressed in brain (Opdam et al., 2000), and Rab6c is expressed in various tissues including brain, testis, prostate and breast (Young et al., 2010). The final Rab protein of this subfamily, Rab41, also termed Rab6d, is the most distant member. It shares more than 60% identity with Rab6 isoforms and within its central portion, amino acids 9 to 192, has almost 80% identity with other members of Rab6 subfamily (Liu et al., 2013).

The role of Rab6a/a' in Golgi organization is rather enigmatic. Neither depletion of Rab6a/a' nor overexpression of GDP-locked form results in disruption of the Golgi ribbon (Jiang and Storrie, 2005; Young et al., 2005; Sun et al., 2007). However, in epistasis studies, Rab6a/a' depletion suppresses Golgi ribbon fragmentation induced by knockdown of Golgi tethers (Sun et al., 2007). In cells treated with siRNA directed against either Zeste White 10 (ZW10)/RINT1 or conserved oligomeric Golgi (COG) retrograde tethers, the Golgi apparatus is fragmented into a cluster of punctate Golgi elements (Hirose et al., 2004; Zolov and Lupashin, 2005; Arasaki et al., 2006; Shestakova et al., 2006; Sun et al., 2007); co-depletion of Rab6a/a' suppresses the ZW10/RINT1- or COG-dependent Golgi disruption (Sun et al., 2007) indicating that retrograde vesicle transport in two separate pathways is Rab-dependent and essential to normal Golgi organization. By electron tomography, Rab6a/a' depletion induces an accumulation of COPI- and clathrin-coated vesicles that is accompanied by an increase in Golgi cisternal number and cisternal continuity (Storrie et al., 2012). These results suggest that Rab6a/a' indirectly regulates Golgi ribbon organization through its effects on Golgi-associated membrane trafficking. In striking contrast, both Rab41/6d depletion and overexpression of GDP-locked Rab41/6d cause Golgi ribbon fragmentation into a cluster of punctate elements, suggesting that Rab41/6d has a direct role in Golgi ribbon organization (Liu et al., 2013). In sum, although both are in the same Rab subfamily, Rab6a/a' and Rab41/6d belong to different functional/phenotypic classes (for review, see Liu and Storrie, 2015).

To explain the different effects of Rab6a/a' and Rab41/6d on Golgi ribbon organization, we identified potential Rab41/6d effectors by yeast two-hybrid assay and characterized their interactions and functional role. After database comparisons, we reduced the yeast two-hybrid hits to 102. None of these hits overlapped with any published Rab6a/a' effectors. Eight were identified as membrane trafficking proteins (UniProtKB search, Echard et al., 1998). Of the 8 proteins, dynactin 6, syntaxin 8, and Kif18A were encoded as full-length protein in the prey plasmid. Our studies show that depletion of dynactin 6 or syntaxin 8, but not Kif18A mimicked the Golgi apparatus phenotype caused by Rab41/6d knockdown, i.e., the Golgi apparatus was disrupted into a cluster of punctate Golgi elements. Co-immunoprecipation experiments further validated the preferential interaction of dynactin 6 and syntaxin 8 with GTP-locked Rab41/6d vs. wild type or GDP-locked Rab41/6d. Therefore, we conclude that dynactin 6 and syntaxin 8 are key Rab41/6d effectors involved in Golgi organization, with dynactin 6, a dynein motor complex subunit, providing a molecular basis for the active role of Rab41/6d in maintaining Golgi ribbon organization.

## Materials and methods

### Cell culture

Cells were grown in a humidified incubator at 37°C and 5% CO_2_. Wild type HeLa cells were cultured in DMEM supplemented with 10% fetal bovine serum (FBS). HeLa cells stably expressing GalNAcT2-GFP were maintained in the presence of 0.45 mg/ml of Geneticin. HEK 293 cells were cultured in DMEM/F-12 supplemented with 10% FBS. Cell culture media, sera, and associated reagents were obtained from Life Technologies, Sigma-Aldrich or Atlas Biologicals.

### Plasmid preparation

cDNA encoding myc-tagged wild type, GTP- or GDP-locked Rab41/6d was prepared as described previously (Liu et al., 2013). cDNA encoding dynactin 6 or syntaxin 8 was synthesized by GeneScript. All constructs were prepared by standard molecular biology techniques. Inserts in all plasmids were validated by sequencing. cDNA encoding myc-tagged wild type, GTP- or GDP-locked Rab6a were gracious gifts from the Ungar laboratory (Department of Biology, University of York, York, UK).

For yeast two-hybrid screening, full-length GTP- or GDP-locked Rab41/6d was digested by BamHI, and then inserted into BamHI site of pGBKT7 bait vector. For co-IP, myc-tagged wild type, GTP- or GDP-locked Rab41/6d was subcloned into pcDNA3.1(+) vector (Life technologies) at the BamHI site. cDNA construct expressing GFP-tagged dynactin 6 or syntaxin 8 protein was generated by inserting dynactin 6 or syntaxin 8 into pEGFP-C1 vector (EcoRI/BamHI) (Clontech).

RUSH plasmids expressing VSVG-GFP or GPI-GFP were gracious gifts from the Perez laboratory (Institut Curie, Paris, France; Boncompain et al., 2012).

### Yeast two-hybrid screening

The GTP-locked Rab41/6d bait construct was transformed into Y2HGold yeast strain. A HeLa cell cDNA library, single copy cDNA enriched, in Y187 yeast strain was obtained from Clontech. Yeast two-hybrid screening was carried out according to the manufacturer's protocol (Clontech). In brief, bait strain was mated with library strain, and the culture was plated on low stringency medium (SD/-Leu/-Trp, DDO) supplemented with X-α-Gal and Aureobasidin A (DDO/X/A) and incubated at 30°C for 3 days. Then all the blue colonies were patched onto high stringency medium (SD/-Leu/-Trp/-His/-Ade, QDO) supplemented with X-α-Gal and Aureobasidin A (QDO/X/A). All QDO/X/A positive hits were further analyzed by PCR amplification followed by HaeIII digestion to eliminate duplicate clones. Positive clones were sequenced using a single primer and then compared to the NCBI database using the BLAST program. Putative functions of the library proteins were identified by searching the UniProtKB database. Eight membrane trafficking and/or vesicle transport proteins were identified and fully sequenced.

### Yeast spotting assay for GTP- vs. GDP-locked Rab41/6d preference

pGBKT7-GTP-Rab41/6d construct or empty vector (negative control) and yeast-rescured, library plasmid were co-transformed into Y2HGold yeast strain. The transformation mix was spread on DDO/X plates and incubated at 30°C for 3 days. Blue colonies were grown in liquid DDO medium to the same optical density (OD_600_ = 0.8) and six 10-fold serial dilutions were prepared. 10 μl of each dilution was spotted on both low stringency (DDO/X/A) and high stringency (QDO/X/A) medium. The growth of yeast was scored after incubating 3 days at 30°C.

### RNA interference

All siRNAs were manufactured by Dharmacon RNA Technologies. The Rab41/6d siRNA [siRab41(4)] sequence has been published previously (Liu et al., 2013). The dynactin 6, syntaxin 8, and Kif18A, siRNA sequences are shown in Table 1. Control siRNAs were siGENOME, non-targeting siRNA #2. According to our previous studies, of the 4 siRNAs directed against Rab41, siRab41(4) caused greatest knockdown and highest level of Golgi disruption (Liu et al., 2013). Thus, siRab41(4) was the selected Rab41/6d siRNA. It was transfected at a final concentration of 200 nM (Liu et al., 2013). All other siRNA duplexes were transfected at a final concentration of 100 nM. Approximately 70, 000 HeLa cells stably expressing Golgi enzyme GalNAcT2-GFP were plated per 35 mm tissue culture dish. After overnight culture, cells were transfected with the corresponding siRNA using DhamaFECT 1 (GE Dharmacon) at the minimum manufacturer's recommended volume of 1 μl/35 mm dish. In order to achieve maximal knockdown, a second cycle of siRNA transfection was done typically 24 h after the initial transfection. Cells were either fixed with formaldehyde or collected for western blotting analysis 96 h after the first transfection cycle. Staining of cells with syntaxin 8 antibody (Synaptic Systems) was performed as described previously (Jiang and Storrie, 2005; Shestakova et al., 2006) to test the knockdown efficiency of syntaxin 8. Staining of cells with rabbit antibodies directed against dynactin 6 (Abcam, 173734) was done with HeLa cells following either formaldehyde or methanol fixation with essentially identical results. Because none of the 4 Kif18A siRNAs exhibited a Golgi phenotypic effect even though all had a substantial to strong effect on HeLa cell viability, we decided not to pursue the question of knockdown level in detail.

**Table 1:** **List of targeted proteins and the siRNAs**.

**Protein**	**siRNA**	**siRNA Sequence**
Kif18A	Kif18A siRNA (1)	GCAAAGAACUUCAGCCUAU
	Kif18A siRNA (2)	UCAAAGAGAUCGAACAUUU
	Kif18A siRNA (3)	GGAGGAAACUGUCAAACUA
	Kif18A siRNA (4)	GCUAUCAGCUCAAACAUAA
Syntaxin 8	Syntaxin 8 siRNA (1)	CACCAAAGCUUACCGUGAC
	Syntaxin 8 siRNA (2)	UCUUGUAACUCGAGAGAGA
	Syntaxin 8 siRNA (3)	GAAUGAGGGUGCCGAACCA
	Syntaxin 8 siRNA (4)	UGAGAUAAUUGACGACCUU
Dynactin 6	Dynactin 6 siRNA (1)	GGACAGUGAUCCACCCUAA
	Dynactin 6 siRNA (2)	CGAAGGGAACCUAAUAGAA
	Dynactin 6 siRNA (3)	UAUCAUAAAUGCUUACCCA
	Dynactin 6 siRNA (4)	AGAUGUAACUAUCGGACCU

### Plasmid transfection for co-immunoprecipitation (co-IP)

HEK 293 cells were transfected at 90% confluence using Lipofectamine 2000 (Life technologies) in Opti-MEM (Life technologies) according to the manufacturer's protocol. After 5 h, an equal volume of DMEM/F-12 containing 10% FBS was added to the transfection medium to achieve 5% FBS final concentration. Cells were harvested 24 h after transfection.

### GFP-binding protein (GBP) bead preparation and co-immunoprecipitation assay

cDNA encoding GBP (Rothbauer et al., 2008) was synthesized by GeneScript and inserted into pET24B vector. Recombinant His6x-tagged GBP was purified on Talon resin (Clontech) and then conjugated to high-density glyoxal agarose beads (Agarose Bead Technologies). GBP beads were used for IP in a similar manner to protein G agarose beads (Ha et al., 2014; Willett et al., 2014, 2015).

HEK 293 cells were transfected as described above. 24 h after transfection, cells were collected and resuspended in lysis buffer (50 mM Tris, pH 7.4, 150 mM NaCl, 1% Triton X-100) supplemented with 10 μl/ml of 100x Halt protease inhibitor cocktail (Pierce) and 2 μl/ml PMSF for 1 h on ice. Cell lysates were centrifuged at 20 000 × g for 10 min, and 90% of the supernatants were added to GBP beads and incubated on a platform rocker at room temperature for 1.5 h. Unbound material was removed by washing the beads three times in 0.05% Triton X-100 in PBS. Proteins bound to the beads were eluted in 2x sample buffer. 10% of the input and 100% of the immunoprecipitate were analyzed by western blotting.

### Western blot analysis

To test the protein knockdown level of dynactin 6, HeLa cells transfected with either control or dynactin 6 siRNA were lysed in 2% SDS, followed by standard SDS-PAGE. Western blotting was performed as described previously (Shestakova et al., 2006; Sun et al., 2007; Majeed et al., 2014) with rabbit polyclonal antibody directed against dynactin 6 (Abcam) and secondary antibody conjugated with IRDye 800 dye (LI-COR). Blots were scanned and analyzed using a LI-COR Odyssey system (LI-COR).

To quantify the co-IP result, samples were run on 4–15% TGX gradient gels (Bio-Rad). Western blotting was performed with a rabbit polyclonal antibody directed against myc (Bethyl), a mouse monoclonal antibody directed against GFP (Covance) and appropriate secondary antibodies conjugated with IRDye 680 or IRDye 800 dyes (LI-COR). Blots were scanned and analyzed using a LI-COR Odyssey system (LI-COR).

### RUSH expression and chase

Wild type HeLa cells were treated with either control siRNA or dynactin 6 siRNA (3) as described above. Typically after 72 h, cells were transfected with RUSH plasmid encoding VSVG-GFP or GPI-GFP using FuGENE HD transfection reagent (Promega) according to the manufacturer's protocol. Expression of RUSH constructs in HeLa cells and chase were performed as described previously with minor modifications (Boncompain et al., 2012). In brief, transfected cells were incubated overnight at 37°C in the presence of avidin (1 × 10^−7^ M, Sigma-Aldrich) for efficient retention of cargo proteins in the ER. After that, 40 μM biotin (Sigma-Aldrich) was added to release the cargo proteins. Cells were incubated at 37°C for various chase (0, 20, 40, 60, 90, and 120 min) in the presence of cycloheximide to prevent further protein synthesis. Cells were then fixed with formaldehyde. Confocal image stacks were taken as described below.

### Fluorescence microscopy and image processing

For antibody staining, HeLa cells stably expressing GalNAcT2-GFP were transfected with corresponding siRNA as described above. 96 h after the first transfection cycle, cells were fixed with formaldehyde and then stained with EEA1 (BD Biosciences), Rab5 (BD Biosciences), M6PR (gift from Bernard Hoflack), LAMP1 (gift from Thomas August), or TGN46 (AbD Serotec) antibody as described previously (Jiang and Storrie, 2005; Shestakova et al., 2006). Confocal image stacks were taken as described below.

For cell surface lectin staining, HeLa cells stably expressing GalNAcT2-GFP were transfected with corresponding siRNA as described above. 96 h after the first transfection cycle, cells were fixed with 1% paraformaldehyde and blocked with 0.1% BSA in PBS. Cells were incubated for 30 min with Alexa Fluor 555 conjugate of WGA lectin (Molecular Probes) diluted in PBS. Confocal image stacks were taken for the analysis of GalNAcT2-GFP distribution, while wide-field images were captured for surface lectin distribution.

Both wide-field images and confocal image stacks were collected with a 63x/1.40 numerical aperture objective and a Zeiss 200M inverted microscope. Confocal image stacks were produced with a BD CARV II spinning disk confocal accessory mounted on the microscope. Images were processed with iVision-MAC software. For the analysis of antibody staining, confocal image stacks were taken at the same exposure time, and the image stacks were condensed into a single plane using a maximum intensity projection protocol (MIP). For the quantification of Golgi fragmentation, confocal images were first deconvolved using Huygens Professional X11 software to sharpen the distinction between Golgi apparatus and general cytoplasmic fluorescence, and then segmented between Golgi fragments and general cytoplasm on the basis of the intensity of GalNAcT2-GFP fluorescence using iVision-Mac software. Number of Golgi fragments was determined based on segmentation using iVision-Mac software. Approximately 30 cells were analyzed for each data point.

## Results

Rab41/Rab6d was identified as a Rab VI subfamily member similar to Rab6a/a' on the basis of homology and structure (Pereira-Leal and Seabra, 2001; Stein et al., 2012). Surprisingly, our previous studies show that manipulation with Rab41/6d and Rab6a/a' expression level have distinctive effects on Golgi organization. By fluorescence microscopy, depletion of Rab6a/a' or overexpression of GDP-locked Rab6a/a' has little, if any, effect on Golgi ribbon organization (Jiang and Storrie, 2005; Young et al., 2005; Sun et al., 2007). In contrast, both Rab41/6d depletion and overexpression of GDP-locked Rab41/6d result in disruption of Golgi ribbon into a cluster of punctate elements (Liu et al., 2013). As a starting point for a mechanistic understanding of this difference, yeast two-hybrid experiment was performed to screen for potential Rab41/6d effectors. We screened a single-copy-enriched, HeLa cell cDNA library by using GTP-locked Rab41/6d as bait. Full-length GTP-locked Rab41/6d was inserted 3′ to the Gal4 DNA-binding domain, while the library cDNA was inserted 3′ to the Gal4 DNA activation domain. In brief, the Y2HGold yeast strain was transformed with GTP-locked Rab41/6d and mated with the Y187 yeast strain transformed with the cDNA library DNA. Colonies containing interacting bait and library fusion proteins were selected by the ability to grow on low stringency medium DDO/X/A. Blue colonies on DDO/X/A were patched onto high stringency medium QDO/X/A. The approximately 5.6 × 10^6^ yeast transformants produced 593 blue colonies on DDO/X/A medium, and among these, 265 displayed the ability to grow on high stringency QDO/X/A agar plates. All QDO/X/A hits were further analyzed by PCR amplification followed by HaeIII digestion to eliminate duplicates containing the same cDNA. 155 non-repetitive hits were obtained and sequenced. Searching the NCBI database revealed that 102 different proteins and protein fragments were obtained (Supplementary Table 1). Of these, 7 were previously shown to be involved in intracellular vesicular trafficking (UniProtKB search, Table 2). Kif18A was also included in Table 2 by analogy with Kif20A, a known Rab6 effector. Kif20A (Rabkinesin-6) interacts with Rab6 and is involved in Rab6-associated intracellular transport (Echard et al., 1998). In brief, of the 102 hits, 8 were identified as putative membrane trafficking effectors of Rab41/6d. Significantly, none of the 102 proteins overlapped with any published Rab6a/a' effector, indicating that, although Rab41/Rab6d and Rab6a/a' are in the same Rab subfamily, they recruit different effectors in regulating Golgi organization.

**Table 2:** **Summary of yeast two-hybrid data and choice of 8 putative Rab41/6d membrane trafficking effectors**.

**(A) Synthetically defined medium selection and PCR screen**		
Low stringency (DDO/X/A)		593 hits
High stringency (QDO/X/A)		265 hits
After duplicate elimination by PCR/HaeIII digestion		155 hits (sequenced using single primer)
**(B) 8 putative Rab41/6d membrane trafficking effectors identified by sequence and database comparisions**		
**Protein name**	**UniProtKB Accession No**.	**Putative functions**
Syntaxin 8	Q9UNK0.2	Vesicle trafficking protein that functions in the early secretory pathway, possibly by mediating retrograde transport from *cis*-Golgi to the ER
Dynactin 6	O00399.1	Antigen processing and presentation of exogenous peptide antigen via MHC class II; mitotic spindle organization
Kif18A	Q8NI77.2	Chromosome congression
Endofin	Q7Z3T8.3	May be involved in regulating membrane trafficking in the endosomal pathway
Adaptor protein complex AP-1 subunit gamma-1	O43747.5	Subunit of clathrin-associated adaptor protein complex 1 that plays a role in protein sorting in the late-Golgi/trans-Golgi network and/or endosomes
Adaptor-related protein complex 3 subunit mu-1	Q9Y2T2.1	Part of the AP-3 complex. The complex facilitates the budding of vesicles from the Golgi membrane and may be directly involved in trafficking to lysosomes
Rabconnectin-3	Q8TDJ6.2	May serve as a scafford protein for both Rab3 GEF and GAP on synaptic vesicles
Selenium-binding protein 1	Q13228.2	May be involved in intra-Golgi protein transport

**Figure 1:**
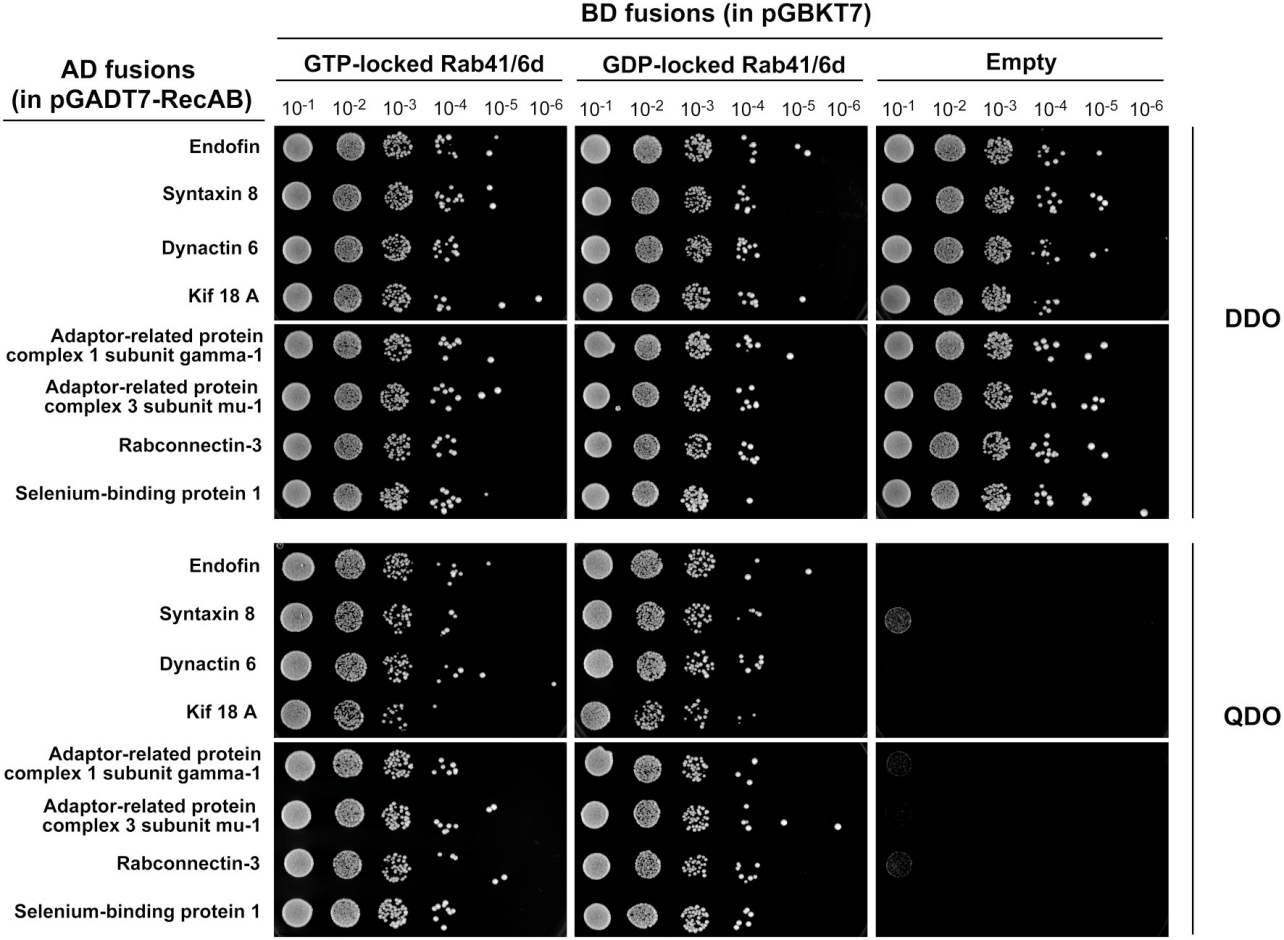
**Interaction of 8 putative effectors with GTP- vs. GDP-locked Rab41/6d evaluated by yeast two-hybrid assay**. Y2HGold yeast cells co-expressing bait (BD fusions) and prey (AD fusions) were grown in liquid DDO medium to the same optical density (OD_600_ = 0.8). Six 10-fold serial dilutions were prepared. 10 μl of each dilution was spotted onto DDO medium, selecting for the presence of both bait and prey plasmids, and QDO medium, selecting for protein interactions.

**Figure 2:**
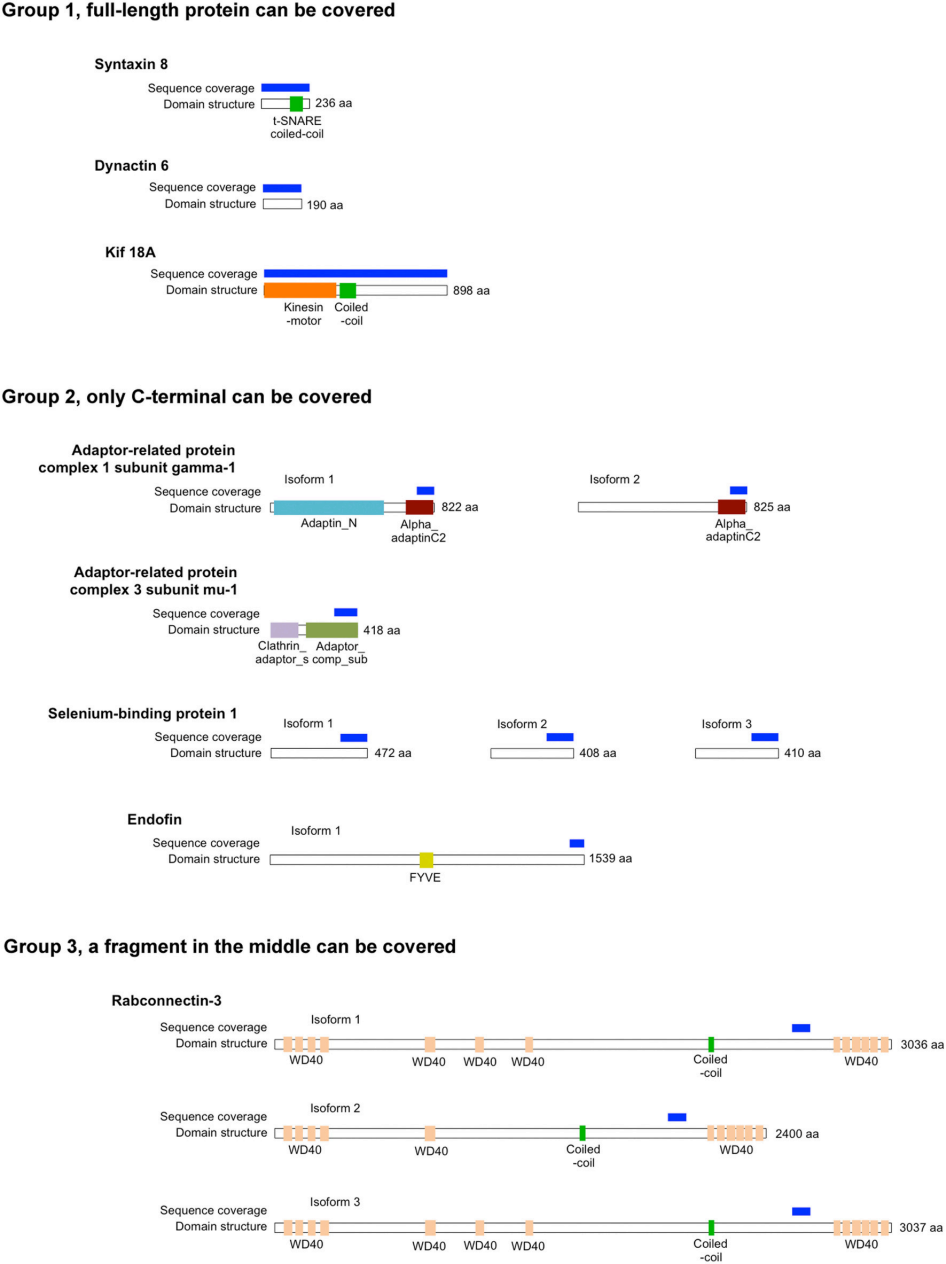
**The length of protein that was identified for 8 putative Rab41/6d effectors by yeast two-hybrid assay**. According to the sequenced cDNA length identified, 8 putative Rab41/6d effectors can be classified into three groups. Dynactin 6, syntaxin 8, and Kif18A, which were sequenced as full-length protein, were in Group 1. Adaptor-related protein complex 1 subunit gamma-1, adaptor-related protein complex 3 subunit mu-1, selenium-binding protein 1 and endofin were in Group 2, and in each of these yeast clone, a fragment at C-terminal was identified. Rabconnectin-3 is the only member of Group 3, with a fragment in the middle portion of the protein was identified.

Rab proteins cycle between an active GTP-bound state and an inactive GDP-bound state. Typically, Rab effectors bind specific Rabs in their GTP-bound states (for review, see Liu and Storrie, 2012). In order to test the preference of these 8 candidate effectors with GTP- vs. GDP-locked Rab41/6d, we carried out directed yeast two-hybrid experiments. For the 8 positive interactions, bait constructs for GTP-, GDP-locked Rab41/6d or empty vector (negative controls) and prey plasmid were co-transformed into Y2HGold yeast strain and spread on DDO medium. Yeast were then grown in liquid DDO medium to the same optical density, serially diluted by 10-fold, and spotted onto DDO medium, selecting for the presence of both bait and prey plasmids, and also QDO medium, selecting for protein interactions. Positive interactions were observed between GTP-locked Rab41/6d and each of the 8 candidate effectors, while interactions with GDP-locked Rab41/6d had less growth, and no interactions were observed with the empty vector, negative control (Figure 1). In sum, the yeast two-hybrid results indicated a preferential interaction of these 8 proteins with GTP-locked Rab41/6d vs. GDP-locked Rab41/6d. These 8 proteins as candidate effectors were further classified into three groups based on the sequenced cDNA length identified in each of the yeast clones. As shown in Figure 2, of the 8 proteins, only dynactin 6 (also known as p27), syntaxin 8 and Kif18A, sequenced as full-length protein, while for all others, only a fragment at either the C-terminal or in the middle portion of the protein was covered in our clones. On the basis of the above analysis, dynactin 6, syntaxin 8 and Kif18A were selected as Rab41/6d candidate effectors for further study.

**Figure 3:**
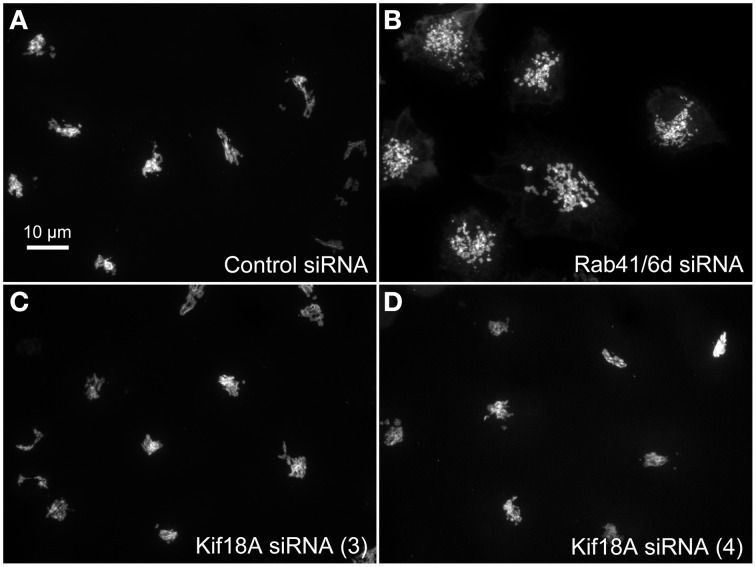
**Kif18A siRNA treatment failed to mimic the Rab41/6d depletion Golgi phenotype**. HeLa cells stably expressing GalNAcT2-GFP were transfected with control siRNA, Rab41/6d siRNA, or Kif18A siRNA and then fixed. Golgi structure was displayed by expression of Golgi enzyme, GalNAcT2-GFP. In contrast with control **(A)**, depletion of Rab41/6d fragmented the Golgi apparatus into a cluster of Golgi elements **(B)**. However, all 4 siRNAs directed against Kif18A had little, if any effect on Golgi ribbon structure **[C, D**, siRNA (3) and (4) outcomes were shown].

**Figure 4:**
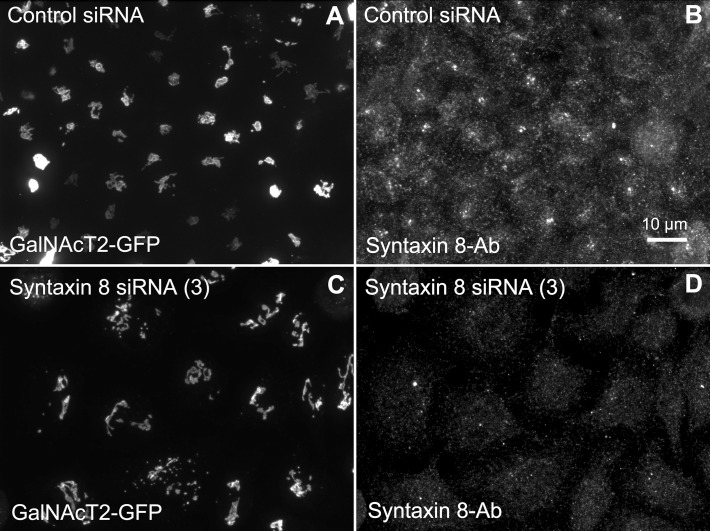
**Syntaxin 8 siRNA treatment weakly mimicked the Rab41/6d depletion Golgi disruption phenotype**. HeLa cells stably expressing GalNAcT2-GFP were transfected with either control or syntaxin 8 siRNA, and then fixed. Golgi structure was displayed by expression of Golgi enzyme, GalNAcT2-GFP. Control cells displayed normal Golgi ribbon structure **(A)**, while cells treated with syntaxin 8 siRNA (3) had weakly clustered punctate Golgi distribution **(C)**. Antibody staining was performed to test the knockdown efficiency of syntaxin 8 siRNA (3). In contrast with control **(B)**, substantial knockdown of syntaxin 8 was observed in syntaxin 8 siRNA (3) treated cells **(D)**.

**Figure 5:**
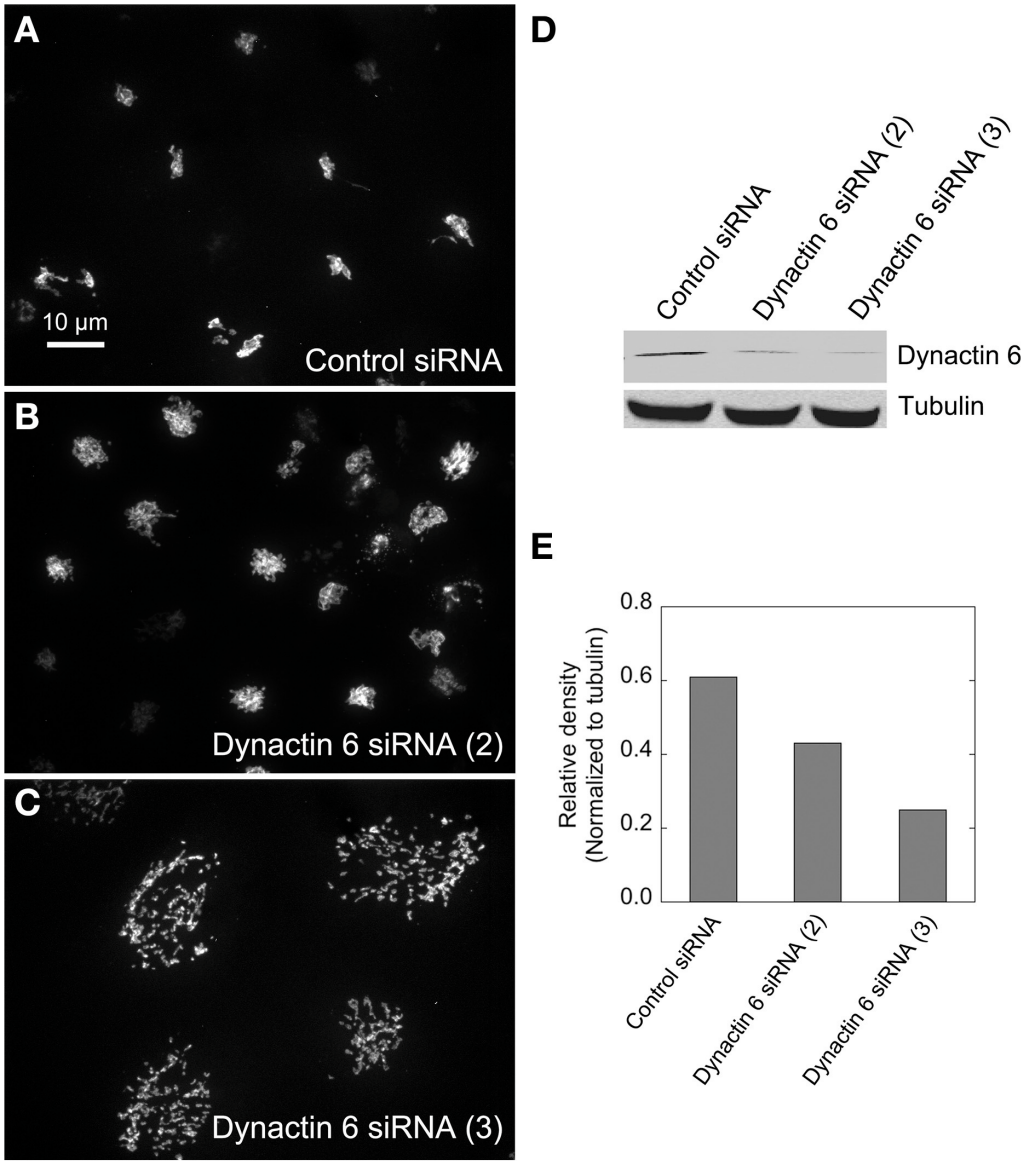
**Dynactin 6 siRNA treatment strongly mimicked the Rab41/6d depletion Golgi disruption phenotype**. HeLa cells stably expressing GalNAcT2-GFP were transfected with either control or dynactin 6 siRNA, and then fixed or collected for western blot analysis. Western blot result shows that both dynactin 6 siRNA (2) and dynactin 6 siRNA (3) caused substantial knockdown of the protein level of dynactin 6 **(D)** with dynactin 6 siRNA (3) gave a higher level of dynactin 6 knockdown **(E)**. Golgi structure was displayed by expression of Golgi enzyme, GalNAcT2-GFP. In contrast with control **(A)**, dynactin 6 siRNA (2) caused a medium level of Golgi disruption **(B)**, while dynactin 6 siRNA (3) was the most effective and caused a high level of Golgi disruption **(C)**.

We hypothesized that dynactin 6, syntaxin 8, and Kif18A were key Rab41/6d effectors involved in regulation of Golgi organization. If so, their individual knockdown should mimic the effect of Rab41/6d-depletion on Golgi organization. As shown in Figure 3 and Table 3, control cells exhibited a relatively compact Golgi ribbon as indicated by the distribution of the stably expressed, tagged Golgi protein, GalNAcT2-GFP. As expected, treatment of HeLa cells with Rab41/6d siRNA fragmented the Golgi ribbon into a clustered punctate Golgi distribution (Liu et al., 2013). In comparison, Kif18A siRNA(3) and (4) had little, if any, effect on juxtanuclear distribution of Golgi apparatus and the other two Kif18A siRNAs tested were prohibitively toxic to HeLa cells, ~70% cells transfected with Kif18A siRNA(1) or (2) died. In contrast, both syntaxin 8 and dynactin 6 siRNA treatments produced a disrupted Golgi apparatus similar to that displayed by Rab41/6d knockdown with dynactin 6 having the stronger effect. As shown in Figure 4, when cells were incubated with syntaxin 8 siRNA, the Golgi apparatus was more weakly fragmented into a cluster of Golgi elements (compare Figures 4C, 5C for quantification, see Table 3). As shown in Figure 5, dynactin 6 knockdown was more pronounced. Dynactin 6 siRNA (2) treatment caused a medium level of Golgi fragmentation (Figure 5B, for quantification, see Table 3), while dynactin 6 siRNA (3) treatment caused a high level of Golgi disruption mimicking that of Rab41/6d knockdown phenotype (Figures 3B, 5C, for quantification, see Table 3). By immunoblotting, we found that treatment of HeLa cells with either dynactin 6 siRNA (2) or (3) caused a substantial knockdown of the protein level of dynactin 6, with the highest level of knockdown corresponding to the strongest phenotype (Figures 5B–E). Our data indicated that siRNA depletion of dynactin 6 or syntaxin 8, but not Kif18A, produced a disrupted Golgi apparatus similar to that displayed by a Rab41/6d knockdown phenotype, i.e., a cluster of punctate Golgi elements, with the effect of dynactin 6 most closely resembling that of Rab41/6d.

**Table 3:** **Quantification of Golgi fragmentation**.

**Protein targeted**	**siRNA**	**Number of Golgi fragments**	**Normalized number of Golgi fragments**
Control	Control siRNA	3.10 ± 0.24	1.0
**Rab41/6d**	**Rab41/6d siRNA**	**34.45 ± 10.39**	**11.1**
Kif18A	Kif18A siRNA (3)	4.21 ± 0.35	1.4
	Kif18A siRNA (4)	3.79 ± 1.41	1.2
Syntaxin 8	Syntaxin 8 siRNA (3)	14.17 ± 3.54	4.6
**Dynactin 6**	Dynactin 6 siRNA (2)	11.28 ± 3.03	3.6
	**Dynactin 6 siRNA (3)**	**27.58 ± 8.05**	**8.9**

Quantification of Golgi fragmentation was determined using deconvolved images to sharpen the distinction between Golgi apparatus and general cytoplasmic fluorescence. Deconvolved images were then segmented between Golgi fragments and general cytoplasm based on the intensity of GalNAcT2-GFP fluorescence, and the number of Golgi fragments was determined using iVision-MAC software. ~30 cells were analyzed for each data point. Data are shown ± standard error of the mean. Bolded data values emphasize the strong Golgi fragmentation with these conditions.

**Figure 6:**
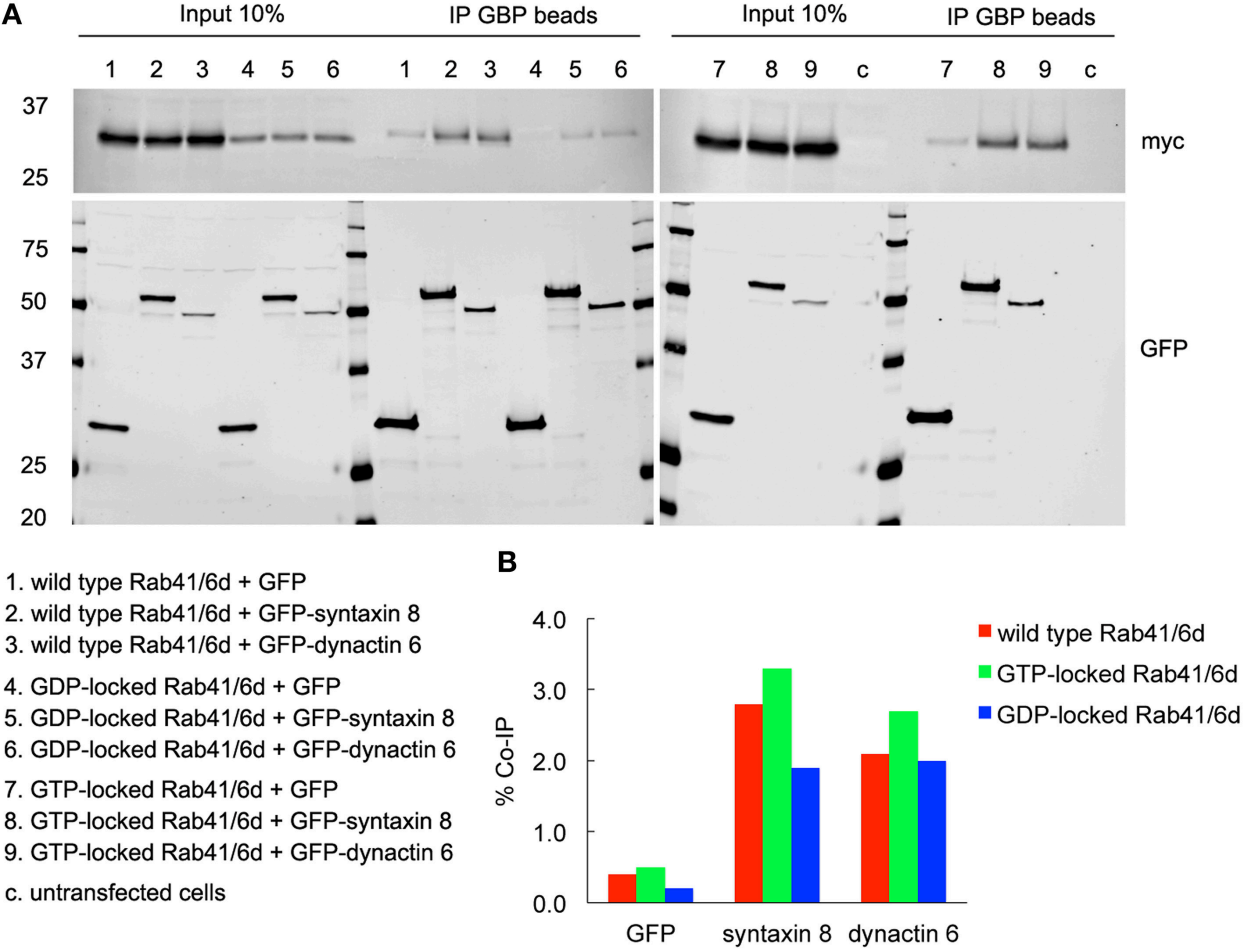
**Both syntaxin 8 and dynactin 6 preferentially bound the GTP-locked form of Rab41/6d over its wild type or GDP-locked form**. HEK 293 cells were transiently co-transfected with plasmids encoding myc tagged wild type, GTP- or GDP-locked Rab41/6d and GFP-syntaxin 8, GFP-dynactin 6, or GFP (as negative control). 24 h after transfection, cells were collected, lysed, and precipitated using agarose beads-conjugated GBP. Immunoprecipitates, along with 10% of total input, were analyzed by 4–15% TGX gradient gel followed by immmunoblotting with antibodies directed against myc (upper panel) and GFP (lower panel) **(A)**. Band densitometry was evaluated by Odyssey software. Co-IP efficiency values were calculated by dividing co-IP by input. The interaction of GTP-locked Rab41/6d with either syntaxin 8 (3.3%) or dynactin 6 (2.7%) was stronger than that of wild type (2.8 and 2.1% co-IP, respectively) and GDP-locked Rab41/6d (1.9 and 2% co-IP, respectively) **(B)**.

**Figure 7:**
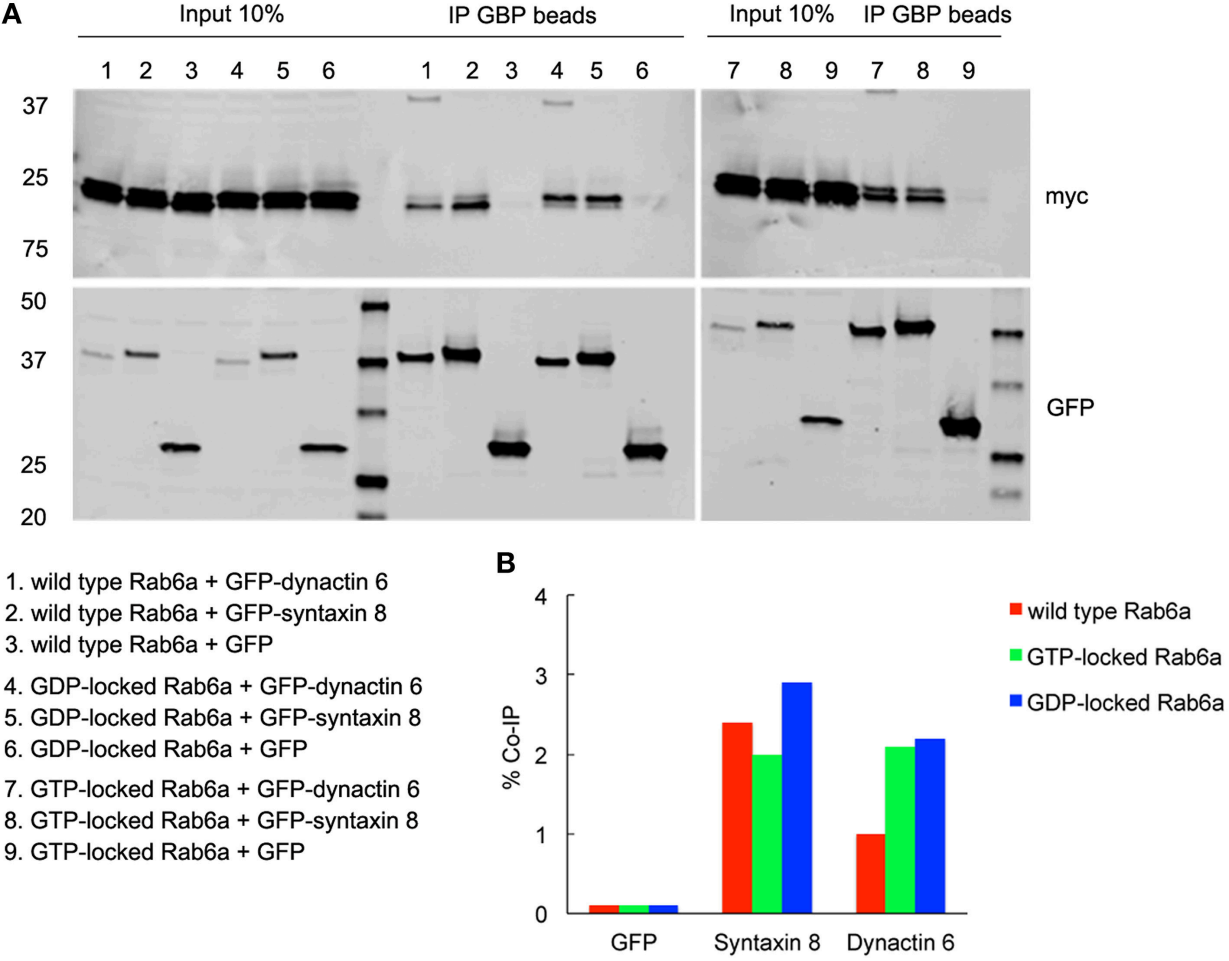
**Both syntaxin 8 and dynactin 6 preferentially bound the GDP-locked form of Rab6a over its wild type or GTP-locked form**. HEK 293 cells were transiently co-transfected with plasmids encoding myc tagged wild type, GTP- or GDP-locked Rab6a and GFP-syntaxin 8, GFP-dynactin 6 or GFP (as negative control). 24 h after transfection, cells were collected, lysed, and precipitated using agarose beads-conjugated GBP. Immunoprecipitates, along with 10% of total input, were analyzed by 4–15% TGX gradient gel followed by immmunoblotting with antibodies directed against myc (upper panel) and GFP (lower panel) **(A)**. Band densitometry was evaluated by Odyssey software. Co-IP efficiency values were calculated by dividing co-IP by input. The interaction of GDP-locked Rab6a with either syntaxin 8 or dynactin 6 was stronger than that of wild type and GTP-locked Rab6a **(B)**.

To validate the interactions of dynactin 6 and syntaxin 8 with GTP-vs. GDP-locked Rab41/6d under cellular conditions, co-immunoprecipation experiments were performed. Myc tagged wild type, GTP-locked or GDP-locked Rab41/6d was co-expressed with GFP-syntaxin 8, GFP-dynactin 6 or GFP (negative control) in HEK 293 cells. As shown in Figure 6A, both GFP-syntaxin 8 and GFP-dynactin 6 differentially immunoprecipitated the three forms of Rab41/6d while immunoprecipitation of GFP, negative control, failed to show interactions. Importantly, we found that the interaction of GTP-locked Rab41/6d with either syntaxin 8 (3.3%) or dynactin 6 (2.7%) was stronger than that of wild type (2.8 and 2.1% co-IP, respectively) or GDP-locked Rab41/6d (1.9 and 2% co-IP, respectively; Figure 6B). Dynactin 6 and syntaxin 8 also immunoprecipted with Rab6a. However, in contrast to Rab41/6d, these two proteins preferentially interacted with the GDP-locked form of Rab6a indicative of a non-physiological interaction (Figure 7). We conclude that both syntaxin 8 and dynactin 6 preferentially bind the GTP-locked form of Rab41/6d over its wild type or GDP-locked form. The co-immunoprecipation together with yeast two-hybrid and knockdown results demonstrates that dynactin 6 and syntaxin 8 are key Rab41/6d effectors involved in Golgi organization. Likely, dynactin 6 as a dynein motor subunit more directly affects Golgi organization, By immunofluorescence dynactin 6 showed a similar distribution to that of dynein (Matanis et al., 2002) in which fluorescence was concentrated in the cytoplasm with focal labeling of peripheral structures (data not shown).

**Figure 8:**
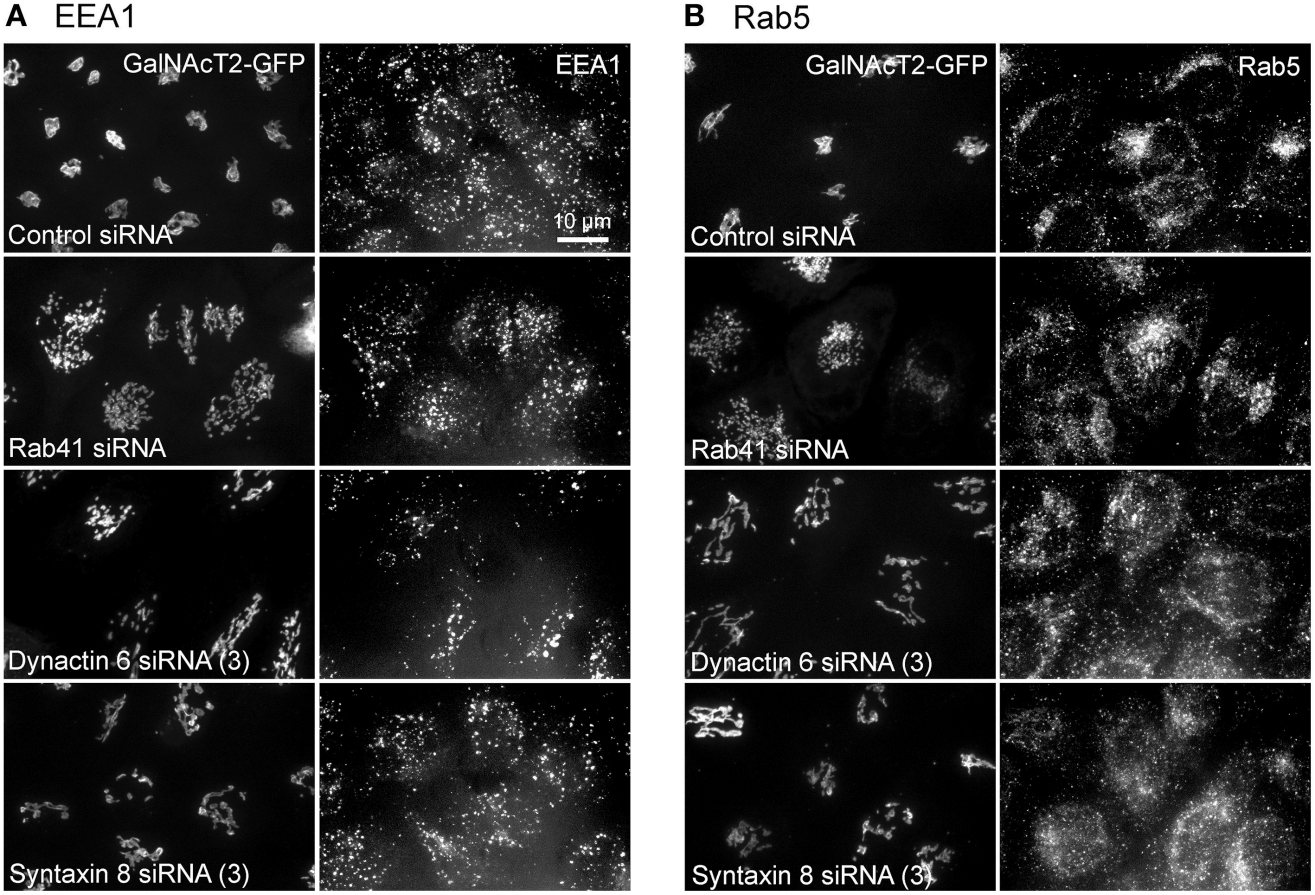
**The distribution of EEA1 and Rab5 showed little change with Rab41, dynactin 6, or syntaxin 8 knockdown**. HeLa cells stably expressing GalNAcT2-GFP were transfected with control, Rab41, dynactin 6, or syntaxin 8 siRNA, and then fixed and stained with EEA1 **(A)** or Rab5 **(B)** antibody. Golgi structure was displayed by expression of Golgi enzyme, GalNAcT2-GFP (left columns in **A**,**B**). In comparison with control cells, Rab41, dynactin 6, or syntaxin 8 siRNA treatment produced little change to the distribution of EEA1 and Rab5 (right columns in **A**,**B**).

**Figure 9:**
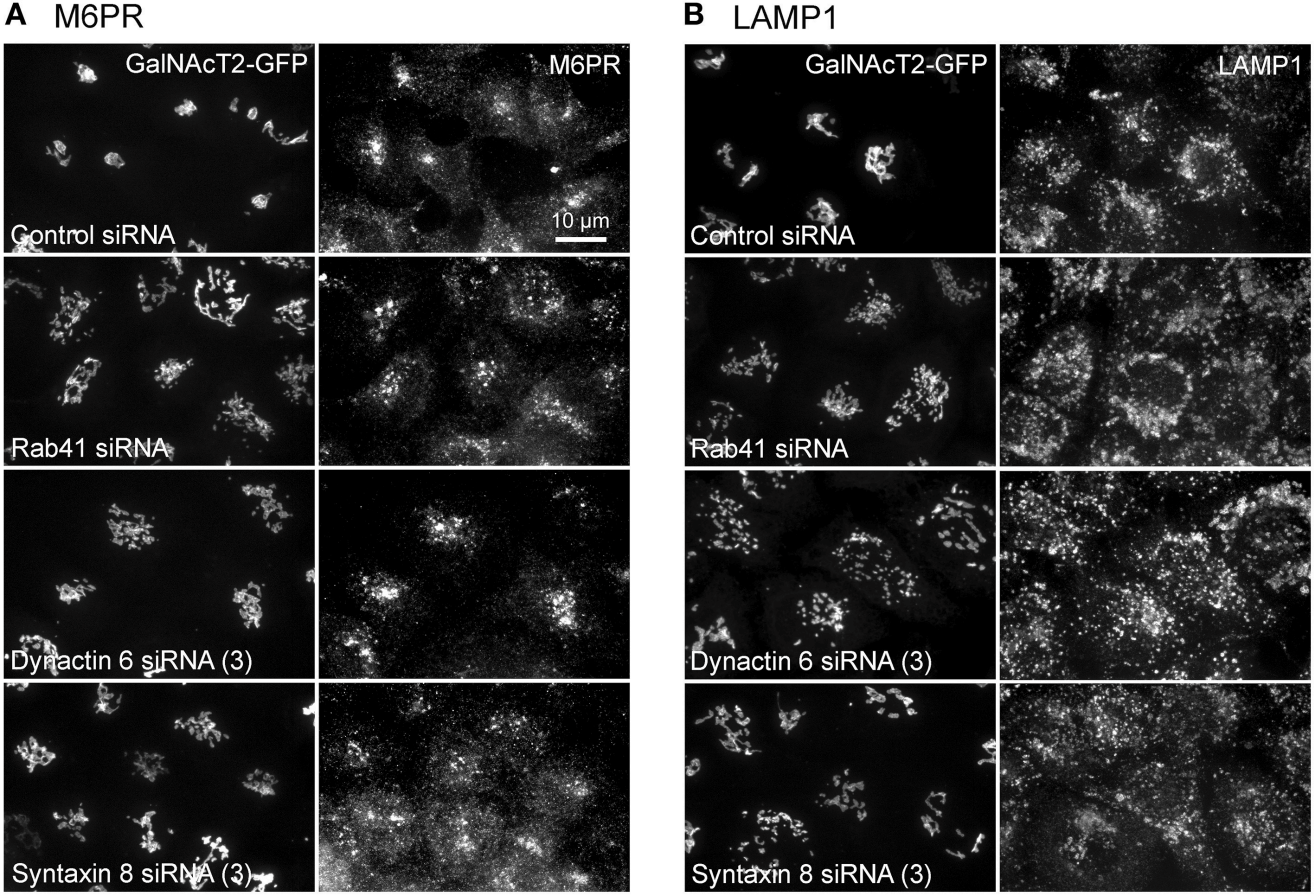
**Depletion of Rab41, dynactin 6 or syntaxin 8 does not affect the distribution of M6PR and LAMP1**. HeLa cells stably expressing GalNAcT2-GFP were transfected with control, Rab41, dynactin 6, or syntaxin 8 siRNA, and then fixed and stained with M6PR **(A)** or LAMP1 **(B)** antibody. Golgi structure was displayed by expression of Golgi enzyme, GalNAcT2-GFP (left columns in **A**,**B**). In comparison with control cells, the distribution of M6PR, and LAMP1 showed little to no change with depletion of Rab41, dynactin 6, or syntaxin 8 (right columns in **A,B**).

**Figure 10:**
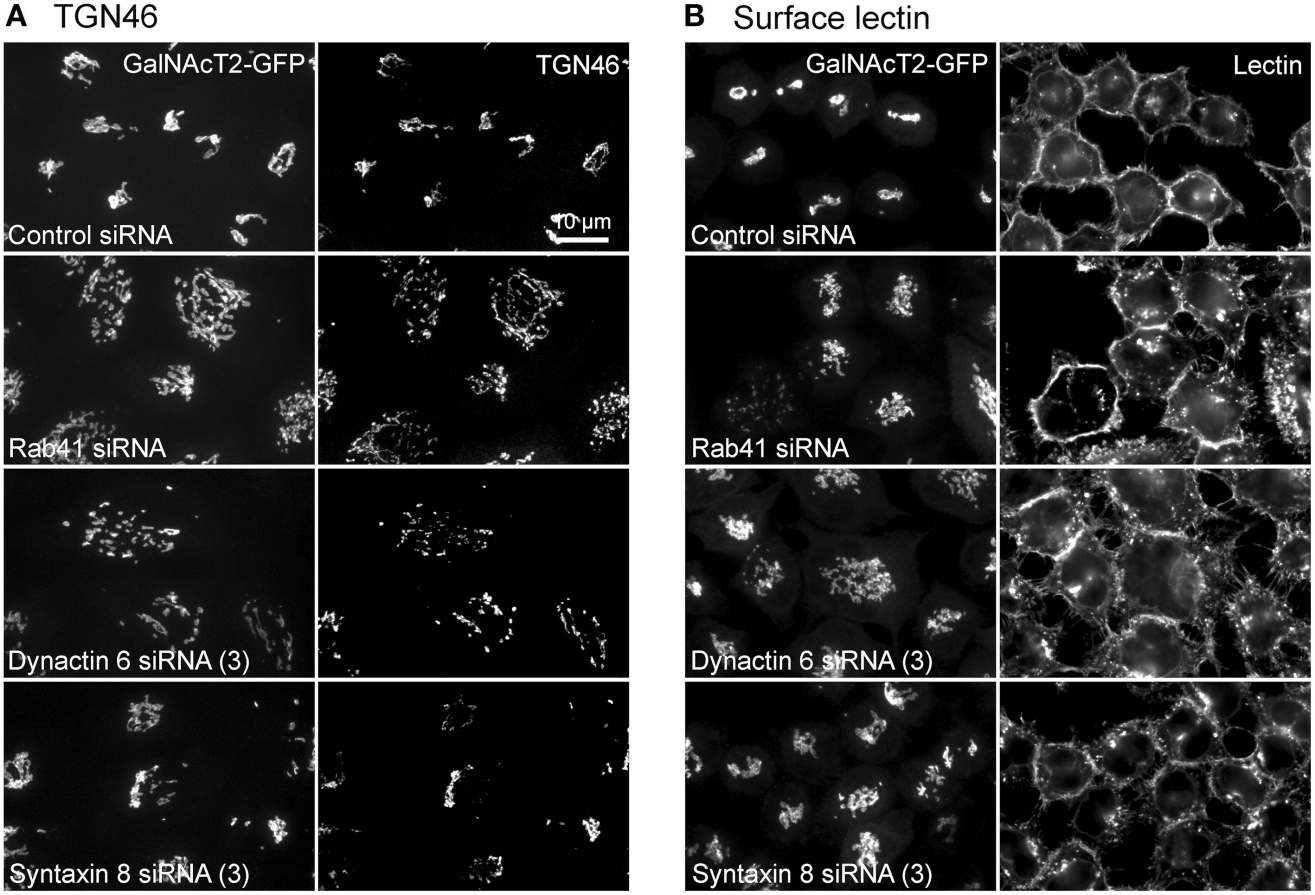
**Rab41, dynactin 6, or syntaxin 8 siRNA treatment produced little change to the level of TGN46 and surface lectin**. HeLa cells stably expressing GalNAcT2-GFP were transfected with control, Rab41, dynactin 6, or syntaxin 8 siRNA, and then fixed and stained with TGN46 antibody **(A)** or Alexa Fluor 555 conjugate of WGA lectin **(B)**. Golgi structure was displayed by expression of Golgi enzyme, GalNAcT2-GFP (left columns in **A** and **B**). In comparison with control cells, Rab41, dynactin 6, or syntaxin 8 knockdown has little effect on the distribution of TGN46 and surface lectin (right columns in **A,B**).

**Figure 11:**
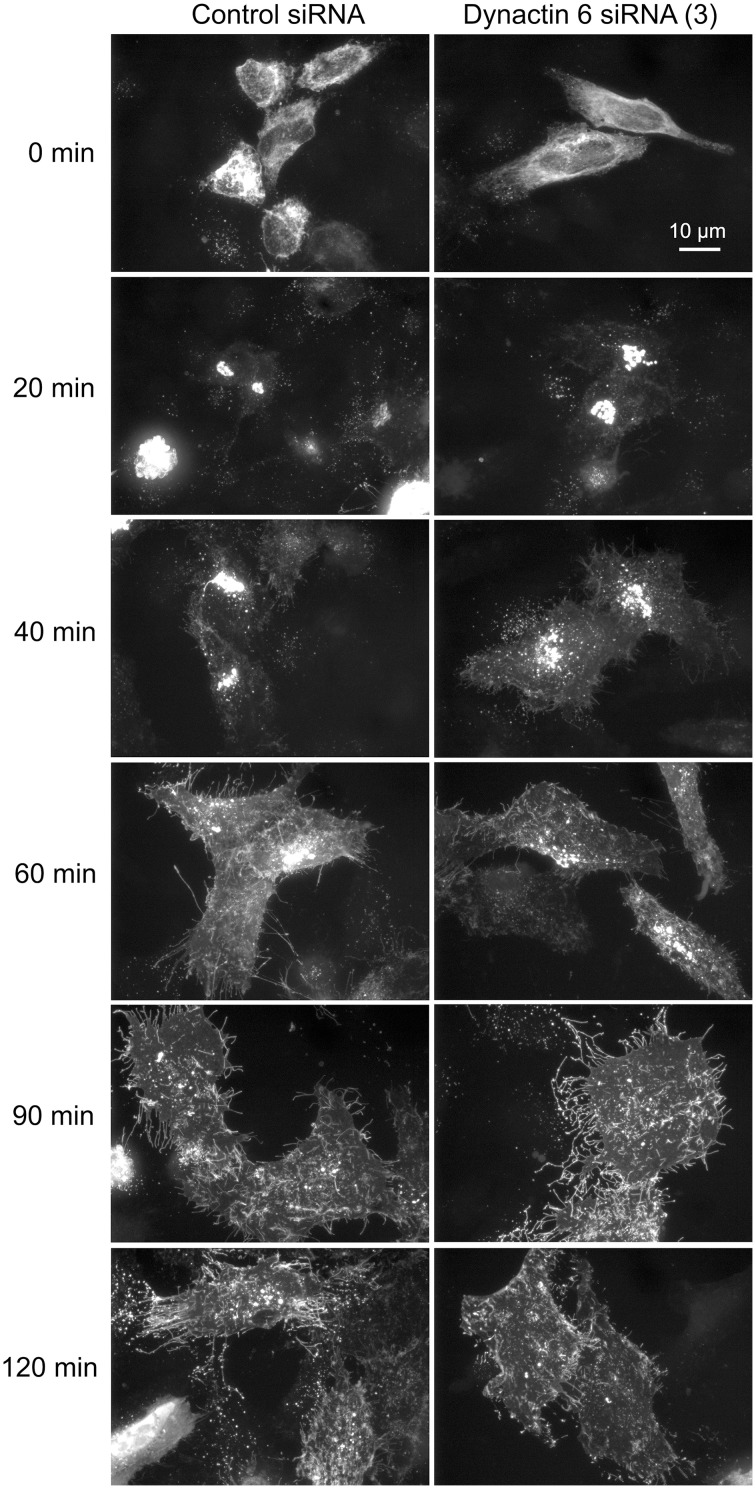
**Depletion of dynactin 6 has little effect on the transport of VSVG-GFP from ER to the Golgi and plasma membrane**. Wild type HeLa cells were incubated with either control siRNA or dynactin 6 siRNA (3) for 72 h and then transfected with RUSH plasmid encoding VSVG-GFP. After 24 h, 40 μM biotin was added. Cells were incubated at 37°C for various chase (0, 20, 40, 60, 90, and 120 min) in the presence of cycloheximide to prevent further protein synthesis. Cells were then fixed and visualized by confocal light microscopy. At 0 min, VSVG-GFP was accumulated in the ER. At the end of a 20-min chase, Golgi accumulation of VSVG was observed. At later chase times, VSVG was transported to the cell surface. With dynactin 6 knockdown, the trafficking of VSVG from ER to the Golgi apparatus and plasma membrane has little to no change. The dispersed nature of the Golgi apparatus in the dynactin 6 siRNA treated cells is especially apparent if the figure is zoomed.

Additionally, we examined the distributions of EEA1/Rab5, endosomal markers, and M6PR/LAMP1, late endosomal/lysosomal markers in Rab41, dynactin 6, or syntaxin 8 knockdown cells. For each, antibodies directed against these proteins were used to stain the cells. Confocal image stacks were taken at the same exposure time, and the stacks were condensed into a single plane in the same manner for control and knockdown cells. The image intensities suggest that depletion of Rab41, dynactin 6, or syntaxin 8 has little to no effect on the distribution of any of these proteins (Figures 8, 9), showing that Rab41, dynactin 6, or syntaxin 8 knockdown effects were Golgi specific. To provide further functional characterization of Rab41 and its two key effectors, dynactin 6 and syntaxin 8, the levels of both TGN46 and surface WGA lectin were examined as described above. As shown in Figure 10, Rab41, dynactin 6, or syntaxin 8 siRNA treatment produced little change in the level of TGN46 or surface lectin staining, indicating that Rab41, dynactin 6, and syntaxin 8 do not have functional role in post-Golgi trafficking pathways. However, our previous results indicate that Rab41 is needed for rapid ER-to-Golgi trafficking (Liu et al., 2013). Therefore, we also tested the involvement of dynactin 6 in ER-to-Golgi and overall ER-to-plasma membrane trafficking using RUSH plasmid expressing VSVG-GFP as cargo protein (Boncompain et al., 2012). In non-treated cells, VSVG-GFP was accumulated in the ER. After the addition of biotin, the cargo protein VSVG-GFP started to move from ER to the Golgi apparatus and plasma membrane. We examined the kinetics of release of VSVG-GFP from ER and found that there was no obvious difference between control cells and dynactin 6 depleted cells (Figure 11). Likely, the transport of another cargo protein, GPI-GFP, from ER to the Golgi and plasma membrane has little change with dynactin 6 knockdown (data not shown). Therefore, we conclude that Rab41 may regulate ER-to-Golgi trafficking through the recruitment of distinct effectors.

## Discussion

The goal of these studies was to establish a molecular basis for the diverse Golgi regulation shown by members of the Rab VI subfamily. For example, Rab6a/a' depletion has minimal direct effect on Golgi ribbon organization at the light microscope level (Young et al., 2005; Majeed et al., 2014), while Rab41/Rab6d depletion results in scattering of the Golgi ribbon into a cloud of punctate Golgi elements (Liu et al., 2013). Taking Rab41/6d as a lead example, we screened by yeast two-hybrid for effectors that could explain the direct role of Rab41/6d in maintenance of the Golgi ribbon (Liu et al., 2013). One hundred and two candidate proteins were identified in the screen and none overlapped with any published Rab6a/a' effectors. Eight were identified as membrane trafficking or vesicle transport proteins (UniProtKB search, Echard et al., 1998). We concentrated on the 3 for which the full-length protein was found in the screen: dynactin 6, syntaxin 8, and Kif18A. Of these, knockdown of dynactin 6 mimicked strongly the Rab41/6d phenotype, i.e., a scattered cluster of Golgi elements, while syntaxin 8 depletion produced a less pronounced phenotype and Kif18A knockdown had little, if any, effect on Golgi ribbon organization. In co-IP experiments, both dynactin 6 and syntaxin 8 appeared to preferentially interact with the GTP-locked form of Rab41/6d indicating that both are bona fide Rab41/Rab6d effectors.

We suggest that dynactin 6, a subunit of the minus-end-directed, dynein-motor complex, a known Golgi positioning protein (for reviews, see Schroer, 2004; Kardon and Vale, 2009; Yadav and Linstedt, 2011) directly affects Golgi organization. Dynactin 6 as a dynactin subunit is involved in most dynein-mediated functions, for example, dynein activation (for review, see King and Schroer, 2000), dynein cargo selection (Holleran et al., 2001) and the localization of dynein to microtubule plus end (Vaughan et al., 2002). Recruitment of cytoplasmic dynein onto Golgi membranes has been shown to be essential for Golgi organization by directly affecting the motor-dependent transport of Golgi elements (for review, see Yadav and Linstedt, 2011). Depletion of cytoplasmic dynein 1 by siRNA produces the scattering of the Golgi ribbon into clustered Golgi elements (Gupta et al., 2008; Majeed et al., 2014). In comparison, syntaxin 8, a member of t-SNARE (soluble N-ethylmaleimide-sensitive factor attachment protein receptor) protein family, likely acts not on Golgi element transport but rather more indirectly through an effect on membrane fusion. Syntaxin 8 forms a complex with syntaxin 7, vti1b, and endobrevin to mediate vesicle trafficking and the fusion of late endosomes (Antonin et al., 2000). We suggest that dynactin 6 is responsible for the active role of Rab41/6d in maintaining Golgi ribbon organization, while syntaxin 8 affects Golgi organization indirectly through an effect on vesicle trafficking/membrane fusion.

In contrast, Rab6a/a'-dependent effects on Golgi organization appear to be an indirect consequence of membrane-trafficking effects. Of the 15 reported Rab6a/a' effectors, only 3, BicD, MyoIIA, and Kif20A, appear to play significant roles in regulating Golgi organization. MyoIIA has been implicated in the fission of Rab6 transport carriers from Golgi membranes and the trafficking of anterograde and retrograde cargo from the Golgi apparatus (Miserey-Lenkei et al., 2010), while Kif20A (also known as rabkinesin 6) is implicated in Golgi-to-ER trafficking (Echard et al., 1998). Knockdown of these motor effectors often produces a more compact Golgi ribbon (Majeed et al., 2014). One of these effectors, BicD1/2, is known to interact with the dynein-dynactin complex and might be expected to have a major role in Golgi organization. BicD1/2 has been shown previously to recruit the dynein-dynactin complex to Golgi membranes and to regulate Golgi-to-ER transport (Matanis et al., 2002; Short et al., 2002). However, BicD1/2 appears, in fact, to have no direct role in Golgi organization. Knockdown of BicD2 has been reported to not alter Golgi ribbon organization (Fumoto et al., 2006) and more detailed analysis indicates that the Golgi ribbon is, if anything, more compact than normal in BicD1/2 depleted HeLa cells (Majeed et al., 2014). We have suggested previously (Majeed et al., 2014) that the interaction of BicD1/2 and dynein/dynactin could be analogous with that of LIS1, another Rab6 effector that forms an idling complex with dynein and arrests dynein motility (Yamada et al., 2013). In sum, Rab41/6d and Rab6a/a' likely have opposite effects on dynein-dependent organization of the Golgi ribbon with Rab41/6d through interaction with dynactin 6 having a direct role in Golgi ribbon organization while Rab6a/a' primarily affects vesicle trafficking with effects on Golgi organization being then indirect.

Despite the shared sequence homology and structural similarity within the Rab VI subfamily, we conclude that the effector sets can be highly divergent between the five subfamily members, Rab6a/a', Rab6b, Rab6c, and Rab41/6d, even when considered in the context of Golgi organization. Although both Rab6a/a' and Rab41/6d play a functional role in Golgi ribbon organization, the effects appear to be opposite to one another. Our yeast two-hybrid screen revealed no common effectors for Rab41/6d and other members of the Rab VI subfamily. Rab6b is preferentially expressed in neuronal cells and implicated in Golgi-associated membrane trafficking (Opdam et al., 2000) and its one reported effector, BicD1/2, is shared with Rab6a/a' (Wanschers et al., 2007). The final family member, Rab6c, has no functional role in membrane trafficking but rather is involved in centrosome duplication and cell cycle progression (Young et al., 2010). Its effectors are unlikely to share any commonality with that of the other subfamily members. In sum, we provide here the first molecular data on the active role of Rab41/6d in maintaining Golgi ribbon organization. In contrast to other family members, Rab41/6d appears to be a direct regulator of Golgi organization through its interaction with dynactin 6.

## Author contributions

SL carried out most of the experiments, analyzed the data and wrote the manuscript. WM tested knockdown of the protein level by antibody staining or western blotting. TK and VL designed and performed co-IP experiments and VL edited portions of the manuscript. BS designed and supported the study, analyzed the data and edited the manuscript.

### Conflict of interest statement

The authors declare that the research was conducted in the absence of any commercial or financial relationships that could be construed as a potential conflict of interest.

## References

[B1] Antonin W. Holroyd C. Fasshauer D. Pabst S. Von Mollard G. F. Jahn R. (2000). A SNARE complex mediating fusion of late endosomes defines conserved properties of SNARE structure and function. EMBO J. 19, 6453–6464. 10.1093/emboj/19.23.6453 11101518 PMC305878

[B2] Antony C. Cibert C. Géraud G. Santa Maria A. Maro B. Mayau V.. (1992). The small GTP-binding protein rab6p is distributed from medial Golgi to the trans-Golgi network as determined by a confocal microscopic approach. J. Cell Sci. 103, 785–796. 1478971 10.1242/jcs.103.3.785

[B3] Arasaki K. Taniguchi M. Tani K. Tagaya M. (2006). RINT-1 regulates the localization and entry of ZW10 to the syntaxin 18 complex. Mol. Biol. Cell 17, 2780–2788. 10.1091/mbc.E05-10-0973 16571679 PMC1474792

[B4] Boncompain G. Divoux S. Gareil N. de Forges H. Lescure A. Latreche L.. (2012). Synchronization of secretory protein traffic in populations of cells. Nat. Methods 9, 493–498. 10.1038/nmeth.1928 22406856

[B5] Echard A. Jollivet F. Martinez O. Lacapère J. J. Rousselet A. Janoueix-Lerosey I.. (1998). Interaction of a Golgi-associated kinesin-like protein with Rab6. Science 279, 580–585. 10.1126/science.279.5350.580 9438855

[B6] Echard A. Opdam F. J. de Leeuw H. J. Jollivet F. Savelkoul P. Hendriks W.. (2000). Alternative splicing of the human Rab6A gene generates two close but functionally different isoforms. Mol. Biol. Cell 11, 3819–3833. 10.1091/mbc.11.11.3819 11071909 PMC15039

[B7] Fumoto K. Hoogenraad C. C. Kikuchi A. (2006). GSK-3beta-regulated interaction of BICD with dynein is involved in microtubule anchorage at centrosome. EMBO J. 25, 5670–5682. 10.1038/sj.emboj.7601459 17139249 PMC1698904

[B8] Goud B. Zahraoui A. Tavitian A. Saraste J. (1990). Small GTP-binding protein associated with Golgi cisternae. Nature 345, 553–556. 10.1038/345553a0 2112230

[B9] Gupta V. Palmer K. J. Spence P. Hudson A. Stephens D. J. (2008). Kinesin-1 (uKHC/KIF5B) is required for bidirectional motility of ER exit sites and efficient ER-to-Golgi transport. Traffic 9, 1850–1866. 10.1111/j.1600-0854.2008.00811.x 18817524

[B10] Ha J. Y. Pokrovskaya I. D. Climer L. K. Shimamura G. R. Kudlyk T. Jeffrey P. D. (2014). Cog5-Cog7 crystal structure reveals interactions essential for the function of a multisubunit tethering complex. Proc. Natl. Acad. Sci. U.S.A. 111, 15762–15767. 10.1073/pnas.1414829111 25331899 PMC4226102

[B11] Hirose H. Arasaki K. Dohmae N. Takio K. Hatsuzawa K. Nagahama M.. (2004). Implication of ZW10 in membrane trafficking between the endoplasmic reticulum and Golgi. EMBO J. 23, 1267–1278. 10.1038/sj.emboj.7600135 15029241 PMC381410

[B12] Holleran E. A. Ligon L. A. Tokito M. Stankewich M. C. Morrow J. S. Holzbaur E. L. (2001). beta III spectrin binds to the Arp1 subunit of dynactin. J. Biol. Chem. 276, 36598–36605. 10.1074/jbc.M104838200 11461920

[B13] Jiang S. Storrie B. (2005). Cisternal rab proteins regulate Golgi apparatus redistribution in response to hypotonic stress. Mol. Biol. Cell 16, 2586–2596. 10.1091/mbc.E04-10-0861 15758030 PMC1087260

[B14] Kardon J. R. Vale R. D. (2009). Regulators of the cytoplasmic dynein motor. Nat. Rev. Mol. Cell Biol. 10, 854–865. 10.1038/nrm2804 19935668 PMC3394690

[B15] King S. J. Schroer T. A. (2000). Dynactin increases the processivity of the cytoplasmic dynein motor. Nat. Cell Biol. 2, 20–24 10.1038/71338 10620802

[B16] Liu S. Hunt L. Storrie B. (2013). Rab41 is a novel regulator of Golgi apparatus organization that is needed for ER-to-Golgi trafficking and cell growth. PLoS ONE 8:e71886. 10.1371/journal.pone.0071886 23936529 PMC3735572

[B17] Liu S. Storrie B. (2012). Are Rab proteins the link between Golgi organization and membrane trafficking? Cell. Mol. Life Sci. 69, 4093–4106. 10.1007/s00018-012-1021-6 22581368 PMC4080914

[B18] Liu S. Storrie B. (2015). How Rab proteins determine Golgi structure. Int. Rev. Cell Mol. Biol. 315, 1–22. 10.1016/bs.ircmb.2014.12.002 25708460 PMC4392918

[B19] Lowe M. (2011). Structural organization of the Golgi apparatus. Curr. Opin. Cell Biol. 23, 85–93. 10.1016/j.ceb.2010.10.004 21071196

[B20] Majeed W. Liu S. Storrie B. (2014). Distinct sets of Rab6 effectors contribute to ZW10–and COG-dependent Golgi homeostasis. Traffic 15, 630–647. 10.1111/tra.12167 24575842 PMC4016170

[B21] Matanis T. Akhmanova A. Wulf P. Del Nery E. Weide T. Stepanova T.. (2002). Bicaudal-D regulates COPI-independent Golgi-ER transport by recruiting the dynein-dynactin motor complex. Nat. Cell Biol. 4, 986–992. 10.1038/ncb891 12447383

[B22] Miserey-Lenkei S. Chalancon G. Bardin S. Formstecher E. Goud B. Echard A. (2010). Rab and actomyosin-dependent fission of transport vesicles at the Golgi complex. Nat. Cell Biol. 12, 645–654. 10.1038/ncb2067 20562865

[B23] Opdam F. J. Echard A. Croes H. J. van den Hurk J. A. van de Vorstenbosch R. A. Ginsel L. A.. (2000). The small GTPase Rab6B, a novel Rab6 subfamily member, is cell-type specifically expressed and localised to the Golgi apparatus. J. Cell Sci. 113, 2725–2735. 10893188 10.1242/jcs.113.15.2725

[B24] Pereira-Leal J. B. Seabra M. C. (2001). Evolution of the Rab family of small GTP-binding proteins. J. Mol. Biol. 313, 889–901. 10.1006/jmbi.2001.5072 11697911

[B25] Rothbauer U. Zolghadr K. Muyldermans S. Schepers A. Cardoso M. C. Leonhardt H. (2008). A versatile nanotrap for biochemical and functional studies with fluorescent fusion proteins. Mol. Cell. Proteomics 7, 282–289. 10.1074/mcp.M700342-MCP200 17951627

[B26] Schroer T. A. (2004). Dynactin. Annu. Rev. Cell Dev. Biol. 20, 759–779. 10.1146/annurev.cellbio.20.012103.094623 15473859

[B27] Shestakova A. Zolov S. Lupashin V. (2006). COG complex-mediated recycling of Golgi glycosyltransferases is essential for normal protein glycosylation. Traffic 7, 191–204. 10.1111/j.1600-0854.2005.00376.x 16420527

[B28] Short B. Preisinger C. Schaletzky J. Kopajtich R. Barr F. A. (2002). The Rab6 GTPase regulates recruitment of the dynactin complex to Golgi membranes. Curr. Biol. 12, 1792–1795. 10.1016/S0960-9822(02)01221-6 12401177

[B29] Stein M. Pilli M. Bernauer S. Habermann B. H. Zerial M. Wade R. C. (2012). The interaction properties of the human Rab GTPase family–comparative analysis reveals determinants of molecular binding selectivity. PLoS ONE 7:e34870. 10.1371/journal.pone.0034870 22523562 PMC3327705

[B30] Storrie B. Micaroni M. Morgan G. P. Jones N. Kamykowski J. A. Wilkins N.. (2012). Electron tomography reveals Rab6 is essential to the trafficking of trans-Golgi clathrin and COPI-coated vesicles and the maintenance of Golgi cisternal number. Traffic 13, 727–744. 10.1111/j.1600-0854.2012.01343.x 22335553 PMC3324626

[B31] Sun Y. Shestakova A. Hunt L. Sehgal S. Lupashin V. Storrie B. (2007). Rab6 regulates both ZW10/RINT-1 and conserved oligomeric Golgi complex-dependent Golgi trafficking and homeostasis. Mol. Biol. Cell 18, 4129–4142. 10.1091/mbc.E07-01-0080 17699596 PMC1995728

[B32] Vaughan P. S. Miura P. Henderson M. Byrne B. Vaughan K. T. (2002). A role for regulated binding of p150(Glued) to microtubule plus ends in organelle transport. J. Cell Biol. 158, 305–319. 10.1083/jcb.200201029 12119357 PMC2173134

[B33] Wanschers B. F. van de Vorstenbosch R. Schlager M. A. Splinter D. Akhmanova A. Hoogenraad C. C.. (2007). A role for the Rab6B Bicaudal-D1 interaction in retrograde transport in neuronal cells. Exp. Cell Res. 313, 3408–3420. 10.1016/j.yexcr.2007.05.032 17707369

[B34] Wei J. H. Seemann J. (2010). Unraveling the Golgi ribbon. Traffic 11, 1391–1400. 10.1111/j.1600-0854.2010.01114.x 21040294 PMC4221251

[B35] Willett R. A. Kudlyk T. A. Lupashin V. V. (2015). Expression of functional Myc-tagged conserved oligomeric Golgi (COG) subcomplexes in mammalian cells. Methods Mol. Biol. 1270, 167–177. 10.1007/978-1-4939-2309-0_13 25702117 PMC4607258

[B36] Willett R. Pokrovskaya I. Kudlyk T. Lupashin V. (2014). Multipronged interaction of the COG complex with intracellular membranes. Cell. Logist. 4:e27888. 10.4161/cl.27888 24649395 PMC3948154

[B37] Yadav S. Linstedt A. D. (2011). Golgi positioning. Cold Spring Harb. Perspect. Biol. 3:a005322. 10.1101/cshperspect.a005322 21504874 PMC3101843

[B38] Yamada M. Kumamoto K. Mikuni S. Arai Y. Kinjo M. Nagai T.. (2013). Rab6a releases LIS1 from a dynein idling complex and activates dynein for retrograde movement. Nat. Commun. 4:2033. 10.1038/ncomms3033 23783758

[B39] Young J. Ménétrey J. Goud B. (2010). RAB6C is a retrogene that encodes a centrosomal protein involved in cell cycle progression. J. Mol. Biol. 397, 69–88. 10.1016/j.jmb.2010.01.009 20064528

[B40] Young J. Stauber T. del Nery E. Vernos I. Pepperkok R. Nilsson T. (2005). Regulation of microtubule-dependent recycling at the trans-Golgi network by Rab6A and Rab6A'. Mol. Biol. Cell 16, 162–177. 10.1091/mbc.E04-03-0260 15483056 PMC539161

[B41] Zolov S. N. Lupashin V. V. (2005). Cog3p depletion blocks vesicle-mediated Golgi retrograde trafficking in HeLa cells. J. Cell Biol. 168, 747–759. 10.1083/jcb.200412003 15728195 PMC2171815

