# Supplementary material for: Identification of Rab41/6d Effectors Provides an Explanation for the Differential Effects of Rab41/6d and Rab6a/a' on Golgi Organization
Source: Front Cell Dev Biol. 2016 Mar 1;4:13. doi: 10.3389/fcell.2016.00013 (PMC4771738; doi:10.3389/fcell.2016.00013)
Supplement: Supplementary file 1 [file Table1.DOC]

Supplementary Material

**Identification of Rab41/6d effectors provides an explanation for the differential effects of Rab41/6d and Rab6a/a'**

**on Golgi organization**

Shijie Liu, Waqar Majeed, Tetyana Kudlyk, Vladimir Lupashin and Brian Storrie*

*** Correspondence:** Corresponding Author: StorrieBrian@uams.edu

**Supplementary Table 1. Rab41/6d-binding proteins identified in yeast two-hybrid screen**

| **Protein name** | **Accession**  **(UniProtKB)** | **Protein length (amino acids)** | **Protein region that is covered^#^** | **Identity of the covered region** |
| --- | --- | --- | --- | --- |
| **Membrane Trafficking/Vesicle Motility Hits with high identity** | | | | |
| Syntaxin 8 | Q9UNK0.2 | 236 | 1-236 | 100% |
| Dynactin 6 | O00399.1 | 190 | 1-190 | 99% |
| KIF18A | Q8NI77.2 | 898 | 1-197, 201-225 | 100% |
| Endofin | Q7Z3T8.3 | 1539 | 1468-1539 | 100% |
| Adaptor protein complex  AP-1 subunit gamma-1 | O43747.5 | 822 | 736-822 | 100% |
| Adapter-related protein complex 3 subunit mu-1 | Q9Y2T2.1 | 418 | 307-346,  344-418 | 100% |
| Rabconnectin-3 | Q8TDJ6.2 | 3036 | 2554-2642 | 100% |
| Selenium-binding protein 1 | Q13228.2 | 472 | 341-471 | 100% |
| **Other Hits with high identity** | | | | |
| WD repeat-containing  protein 61 | Q9GZS3.1 | 305 | 13-234 | 100% |
| Phosphoglucomutase-1 | P36871.3 | 562 | 456-562 | 99% |
| Cyclophilin E | Q9UNP9.1 | 301 | 1-184 | 95% |
| Cyclophilin A | P62937.2 | 165 | 1-24, 22-165 | 99% |
| Sodium/potassium-transporting ATPase  subunit beta-3 | P54709.1 | 279 | 135-279 | 100% |
| Sodium/potassium-transporting ATPase  subunit beta-1 | P05026.1 | 303 | 114-303 | 100% |
| PNN-interacting serine/arginine-rich protein | Q8TF01.2 | 805 | 641-738 | 100% |
| Protein FAM35A | Q86V20.1 | 835 | 332-482 | 100% |
| BTB/POZ domain-containing protein 6 | Q96KE9.3 | 485 | 376-485 | 100% |
| COMM domain-containing protein 1 | Q8N668.1 | 190 | 10-190 | 100% |
| Centrosomal protein of  192 kDa | Q8TEP8.2 | 1941 | 1857-1941 | 99% |
| UMP-CMP kinase | P30085.3 | 196 | 52-196 | 100% |
| Angiogenic factor with G patch and FHA domains 1 | Q8N302.2 | 714 | 465-684,  679-711 | 98% |
| Methionine synthase reductase | Q9UBK8.3 | 725 | 229-380,  408-516 | 99% |
| Fas apoptotic inhibitory molecule 1 | Q9NVQ4.1 | 179 | 24-179 | 100% |
| Tight junction protein 1 | Q07157.3 | 1748 | 1521-1748 | 97% |
| Glutathione-dependent formaldehyde dehydrogenase | P11766.4 | 374 | 1-55, 57-252, 243-311 | 96% |
| Peroxiredoxin-6 | P30041.3 | 224 | 138-224 | 100% |
| Methyltransferase-like  protein 5 | Q9NRN9.1 | 209 | 73-180, 198-209 | 99% |
| Histone deacetylase 2 | Q92769.2 | 488 | 304-488 | 99% |
| Protein timeless homolog | Q9UNS1.2 | 1208 | 1119-1186 | 100% |
| **Most common false positives** (for review, see Stephens and Banting, 2000) | | | | |
| COP9 signalosome complex subunit 5 | Q92905.4 | 334 | 44-283 | 98% |
| Proteasome subunit alpha type-6 | P60900.1 | 246 | 82-246 | 100% |
| Proliferating cell nuclear antigen | P12004.1 | 261 | 178-260 | 100% |
| Electron transfer flavoprotein subunit alpha, mitochondrial | P13804.1 | 333 | 246-333 | 100% |
| Outer mitochondrial membrane protein porin 2 | P45880.2 | 294 | 175-294 | 100% |
| 39S ribosomal protein L36, mitochondrial | Q9P0J6.1 | 103 | 1-103 | 100% |
| Succinate dehydrogenase [ubiquinone] iron-sulfur subunit, mitochondrial; | P21912.3 | 280 | 77-280 | 99% |
| Propionyl-CoA carboxylase alpha chain, mitochondrial | P05165.4 | 728 | 539-548,  581-616 | 94% |
| Serine beta-lactamase-like protein LACTB, mitochondrial | P83111.2 | 547 | 453-547 | 99% |
| Pyruvate dehydrogenase protein X component, mitochondrial | O00330.3 | 501 | 293-501 | 99% |
| Elongation factor G 2, mitochondrial | Q969S9.1 | 779 | 583-779 | 99% |
| Heterogeneous nuclear ribonucleoprotein A/B | Q99729.2 | 332 | 320-332 | 100% |
| Heterogeneous nuclear ribonucleoprotein methyltransferase-like  protein 3 | O60678.3 | 531 | 464-531 | 97% |
| Small nuclear ribonucleoprotein G | P62308.1 | 76 | 1-76 | 100% |
| Ribosomal L24 domain-containing protein 1 | Q9UHA3.1 | 163 | 146-163 | 100% |
| 60S ribosomal protein L12 | P30050.1 | 165 | 1-13, 42-165 | 100% |
| Translation initiation factor  IF-2 | O60841.4 | 1220 | 1100-1220 | 99% |
| ER-associated Hsp40  co-chaperone | Q9UBS4.1 | 358 | 255-358 | 98% |
| Heat shock protein 40 | P25685.4 | 340 | 199-336 | 96% |
| Heat shock protein J2 | P31689.2 | 397 | 116-350 | 99% |
| Heat shock 70 kDa protein 5 | P11021.2 | 654 | 433-654 | 99% |
| Threonyl-tRNA synthetase | P26639.3 | 723 | 601-723 | 81% |
| Arginyl-tRNA--protein transferase 1 | O95260.2 | 518 | 149-319 | 81% |
| Zinc finger protein 32 | P17041.2 | 273 | 138-273 | 100% |
| Isopentenyl pyrophosphate isomerase 1 | Q13907.2 | 227 | 49-227 | 100% |
| Protein phosphatase 1 regulatory subunit 3C | Q9UQK1.2 | 317 | 209-317 | 100% |
| Ubiquitin-fold modifier-conjugating enzyme 1 | Q9Y3C8.3 | 167 | 45-167 | 100% |
| Inactive ubiquitin-specific peptidase 50 | Q70EL3.1 | 339 | 318-339 | 100% |
| Ubiquitin-conjugating  enzyme E2N | P61088.1 | 152 | 1-74, 74-152 | 100% |
| **Hits with low identity** | | | | |
| Homeobox protein DLX-6 | P56179.2 | 175 | 5-75 | 28% |
| Myosin light chain kinase family member 4 | Q86YV6.2 | 388 | 213-269 | 32% |
| Glutamate receptor ionotropic | O60391.2 | 1043 | 540-623 | 26% |
| UDP-N-acetylhexosamine pyrophosphorylase | Q16222.3 | 522 | 411-435 | 48% |
| Olfactory receptor 11L1 | Q8NGX0.1 | 322 | 186-227 | 38% |
| Zinc finger protein 277 | Q9NRM2.2 | 450 | 360-408 | 31% |
| Zinc finger protein 649 | Q9BS31.1 | 505 | 437-501 | 26% |
| Zinc finger and BTB domain-containing protein 49 | Q6ZSB9.3 | 765 | 403-425 | 50% |
| MCM3-associated gene antisense protein | Q9NVU1.1 | 122 | 62-102 | 39% |
| Protein MOST-1 | Q9NRJ1.1 | 99 | 71-98 | 62% |
| RNA-binding protein  with multiple splicing | Q93062.1 | 196 | 167-196 | 43% |
| Kelch-like protein 32 | Q96NJ5.2 | 620 | 383-426 | 38% |
| Dynein heavy chain 3, axonemal | Q8TD57.1 | 4116 | 3294-3335 | 27% |
| Myosin heavy chain 14 | Q7Z406.2 | 1995 | 1913-1965 | 30% |
| Mediator of RNA polymerase II transcription subunit 13 | Q9UHV7.3 | 2174 | 115-144 | 33% |
| Oxidative stress-induced growth inhibitor 1 | Q9UJX0.3 | 560 | 182-219 | 38% |
| MCM3-associated gene antisense protein | Q9NVU1.1 | 122 | 28-100 | 25% |
| Protein FAM227A | F5H4B4.1 | 570 | 470-490 | 57% |
| Protein FAM160A1 | Q05DH4.2 | 1040 | 496-527 | 36% |
| Protein FAM138A/B/C/F | Q6VEP3.1 | 85 | 14-44 | 68% |
| Abhydrolase domain-containing protein 15 | Q6UXT9.2 | 468 | 361-388 | 43% |
| Zinc finger DHHC domain-containing protein 8 | Q9ULC8.3 | 765 | 114-137,  167-205 | 42% |
| Band 4.1-like protein 5 | Q9HCM4.3 | 733 | 152-223 | 31% |
| YTH domain family protein 3 | Q7Z739.1 | 585 | 96-122 | 37% |
| OTU domain-containing protein 4 | Q01804.3 | 1113 | 48-72 | 40% |
| Metabotropic glutamate receptor 5 | P41594.2 | 1212 | 198-266 | 22% |
| Ubiquitin-conjugating enzyme E2 L3 | P68036.1 | 154 | 1-86, 49-154 | 73% |
| E3 ubiquitin-protein ligase HUWE1 | Q7Z6Z7.3 | 4374 | 45-61 | 59% |
| E3 ubiquitin-protein ligase RNF43 | Q68DV7.1 | 783 | 210-268 | 26% |
| E3 ubiquitin-protein ligase Praja-2 | O43164.4 | 708 | 376-402 | 37% |
| A disintegrin and metalloproteinase with thrombospondin motifs 6 | Q9UKP5.2 | 1117 | 228-255 | 52% |
| Uncharacterized protein C14orf178 | Q8N769.1 | 122 | 35-54 | 80% |
| Uncharacterized protein C11orf72 | Q8NBR9.1 | 251 | 89-180 | 28% |
| ATP-binding cassette sub-family C member 3 | O15438.3 | 1527 | 134-191 | 34% |
| Protein-glutamine gamma-glutamyltransferase 2 | P21980.2 | 687 | 539-567 | 34% |
| Inner nuclear membrane protein Man1 | Q9Y2U8.2 | 911 | 622-638 | 71% |
| Brain-specific angiogenesis inhibitor 3 | O60242.2 | 1522 | 319-360 | 37% |
| Serine/threonine-protein kinase SIK3 | Q9Y2K2.3 | 1263 | 193-242 | 38% |
| Down syndrome critical region protein 4 | P56555.1 | 118 | 58-90 | 36% |
| ATP-binding domain-containing protein 4 | Q7L8W6.3 | 267 | 195-208 | 64% |
| Outer dense fiber protein 3 | Q96PU9.1 | 254 | 82-130 | 35% |
| Cleavage and polyadenylation-specific factor 3-like protein | Q5TA45.2 | 600 | 312-390 | 28% |
| Mitogen-activated protein kinase kinase kinase kinase 2 | Q12851.2 | 820 | 539-580 | 36% |
| Follistatin-related protein 5 | Q8N475.2 | 847 | 710-743 | 29% |

^#^The first 8 clones were fully sequenced, while all the others were sequenced using single primer
